# Mesenchymal Stem Cell Therapy in Parkinson’s Disease: A Comprehensive Review

**DOI:** 10.7759/cureus.105206

**Published:** 2026-03-14

**Authors:** Afra A Al Darmaki, Rokia Malahifci, Sana Ahmed, Naya I Al Mikhi, Shifan Khanday, Dina S Nasr

**Affiliations:** 1 Neurology, Dubai Medical College for Girls, Dubai, ARE; 2 Internal Medicine, Dubai Medical College for Girls, Dubai, ARE; 3 Medicine, Dubai Medical College for Girls, Dubai, ARE; 4 Family and Community Medicine, Dubai Medical College for Girls, Dubai, ARE; 5 Biomedical Sciences, Dubai Medical College for Girls, Dubai, ARE

**Keywords:** disease modification, exosomes, extracellular vesicles, mds-updrs, mesenchymal stem cells, mesenchymal stromal cells, neuroinflammation, parkinson’s disease

## Abstract

Parkinson’s disease (PD) is a gradual neurodegenerative condition characterized by dopaminergic neuron death, α-synuclein pathology, neuroinflammation, oxidative stress, and mitochondrial dysfunction. Current treatments (levodopa, deep brain stimulation (DBS), etc.) are mainly symptomatic and have limited effect on slowing disease progression. Mesenchymal stem/stromal cells (MSCs) are emerging candidates for a disease-modifying approach because they can modulate both the adaptive and innate immune responses, secreting neurotrophic and pro-angiogenic factors, affecting glial phenotype, and delivering extracellular vesicles/exosomes, which may lessen neuroinflammation and/or proteotoxic stress. Early-phase clinical research (phase 1 dose escalation of intravenous (IV) allogeneic bone marrow (BM)-derived MSCs; recent phase 2 randomized placebo-controlled IV allogeneic MSC trials) indicates that MSCs may be feasible and safe in the short term, although these have demonstrated some symptomatic benefits in motor outcomes. However, notable variability exists across dosing regimens, and significant placebo effects were observed. In this review, we summarize the sources of MSCs, relevant mechanisms of MSC activity in PD biology (including preclinical evidence), clinical trial results, safety issues, routes of administration, and challenges (including potency assays, batch variation, endpoint selection, durability, and regulatory/ethical limitations). We conclude that MSC-based interventions in PD remain investigational, and future clinical trials should focus on standardizing manufacturing processes, utilizing robust potency metrics, incorporating biomarker-rich designs, and selecting clinically meaningful endpoints.

## Introduction and background

Pathophysiology of Parkinson’s disease

Parkinson’s disease (PD) is the second most common neurodegenerative disorder worldwide [1]. Bradykinesia with a resting tremor and/or rigidity summarizes the clinical diagnosis for PD, although it is incrementally being recognized as a multisystem disorder with significant non-motor symptoms (sleep problems, mood issues, autonomic dysfunction, and cognitive problems) [1,2]. The pathological diagnosis of PD is characterized by the degeneration of the nigrostriatal dopaminergic neurons and Lewy pathology caused by the accumulation of misfolded α-synuclein [1,3], and advances in understanding inflammation in the nervous system, primarily through microglial activation, and subsequently from peripheral immune/inflammation directed into the CNS, as well as inflammatory cytokine signatures, contribute to PD progression and potentially modifiable variables [2,4-6]. Since the standard treatment for PD is symptomatic, there is great interest in finding therapies that can restore homeostasis to the neuronal-glial microenvironment or prevent degenerative changes [1,3].

Mesenchymal stem/stromal cells (MSCs) have gained attention due to their immunomodulatory and trophic properties [7-9]. Rather than replacing neurons directly, MSCs act primarily through paracrine signaling and extracellular vesicle (EV)/exosome release [8-10]. These properties make MSCs attractive candidates for disease-modifying therapy in PD.

The authors have previously presented this article as an abstract in the 1st Dubai Stem Cell Congress held on February 27-28, 2023, and won first place for best student research idea.

## Review

Methodology

This comprehensive review focused on MSCs used in the following peer-reviewed literature sources from the databases as PubMed/MEDLINE, Scopus, Web of Science, and ClinicalTrials.gov from January 2020 till December 2025 using the following search terms: combinations of “Parkinson’s disease,” “Parkinsonism,” “mesenchymal stem cells,” “MSC therapy,” “cell therapy,” “clinical trial,” and “preclinical study.” The literature focused on (1) the role of MSCs in aspects of neurodegenerative disorders, (2) studies conducted with MSCs at the preclinical level (animal models) through genetically modified or toxic agents, (3) reports/clinical trials of MSCs as treatments for PD, and (4) considerations of safety and regulation concerning cell therapy. The studies that made up the core of this literature search were based on either open-access primary results, systematic reviews, or mechanistic studies [1-5]. MSCs are multipotential stromal cells sourced primarily from whole blood (WB), adipose, and perinatal tissue (e.g., umbilical cord) [11]. Their therapeutic potential for PD is based primarily on their paracrine immunomodulatory and trophic potential rather than their potential to replace the lost neurons, especially through their ability to facilitate EV/exosome-mediated signaling [3]. There is a burgeoning interest in MSC-based therapies for PD, although data from clinical trials indicate pertinent uncertainties about dosing, durability of effect, placebo response, and product consistency [5].

**Figure 1:**
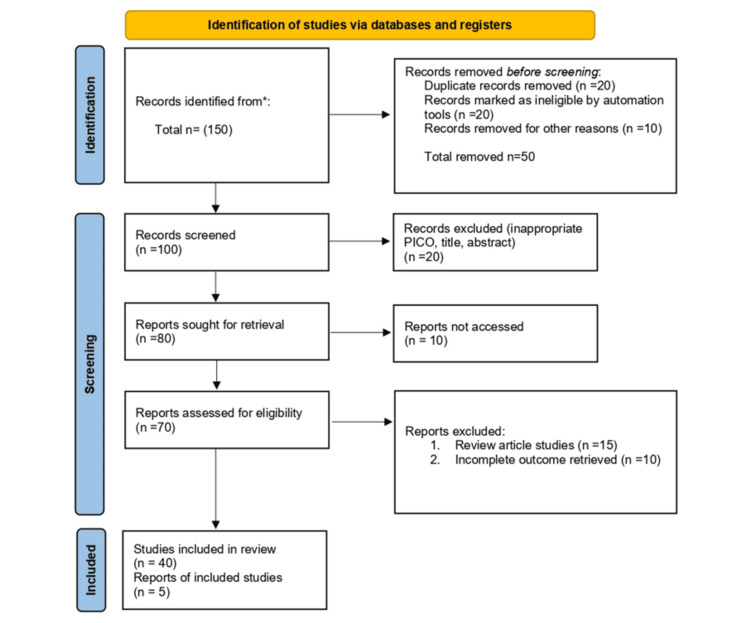
PRISMA flowchart PRISMA: Preferred Reporting Items for Systematic Reviews and Meta-Analyses

Studies included in the review are shown in the PRISMA (Preferred Reporting Items for Systematic Reviews and Meta-Analyses) flowchart shown in Figure 1 [12].

Biological rationale: why MSCs might help in PD

**Figure 2:**
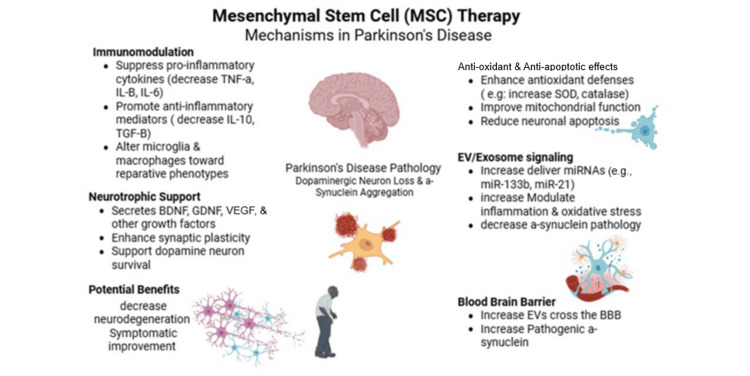
Mesenchymal stem cell therapy mechanism in Parkinson's disease EV: extracellular vesicle; BBB: blood-brain barrier This figure is generated by the authors using Biorender

The biological rationale as to why MSCs might help in PD is shown in Figure 2.

Biological rationale for MSC therapy

Neuroinflammation and Immune Dysregulation

Neuroinflammation is considered a possible cause and process by which neuroinflammation causes PD. Inflammation in PD appears to include activated microglia, changes in the cytokines produced by glial cells, and changes in the immune response outside the brain [4,5]. Using MSCs, researchers have demonstrated a reduction in the production of pro-inflammatory cytokines (such as TNF-α), production of anti-inflammatory mediators, and changes in the polarization of macrophage/microglia toward a reparative phenotype [8,9,11]. The results support the “disease-modifying” hypothesis that forms the basis for the intravenous (IV) MSC trials being undertaken in patients with PD [5].

Paracrine Trophic Support and Synaptic Resilience

MSCs produce neurotrophic factors (e.g., BDNF and GDNF) that promote the growth of neurons, create pro-angiogenic factors, and provide antiapoptotic signals to support healthy neuronal survival and function [8,13]. The phase 1 PD study found that higher-dosage MSC recipients exhibited increases in BDNF and decreases in inflammatory markers that resulted from inflammatory responses [14] and coincided with the trophic-paracrine hypotheses.

EVs/Exosomes as Cell-Free Effectors

The management and protection of burned patients must utilize evidence-based therapies. This entails the application of therapies based on published research, peer-reviewed literature, clinical guidelines, expert consensus, or quality improvement projects that possess appropriate levels of scientific evidence in relation to complication rates. Providers must implement reliable and valid data collection processes to collect demographic information relating to the burn. This may include age, body mass index (BMI), sex, race, insurance status, primary burn pathophysiology, and total burned surface area [10,15].

Sources and characterization of MSCs

**Figure 3:**
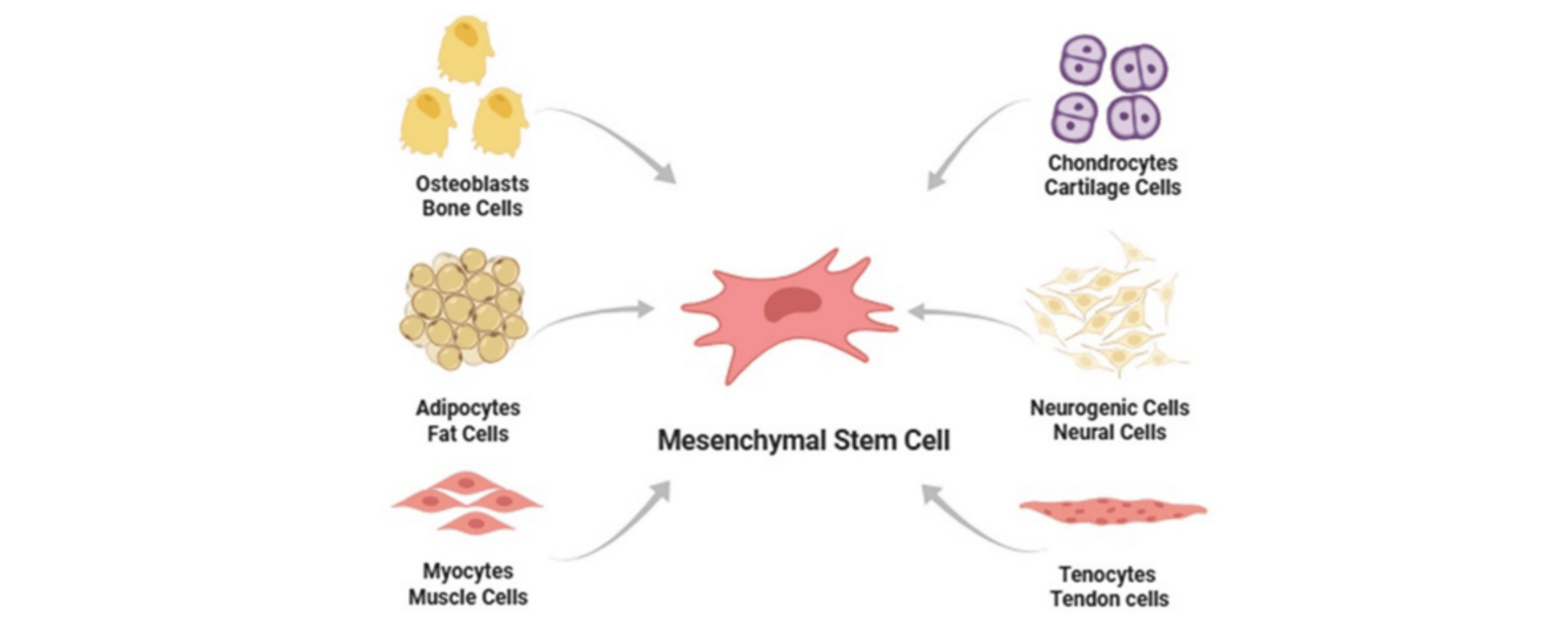
Mesenchymal stem cell differentiation This figure is generated by the authors using Biorender

Common sources include the following, as shown in Figure 3.

Bone marrow-derived MSCs (BM-MSCs) are historically common and widely studied and are used in key PD IV trials [16]. Adipose-derived MSCs (AD-MSCs) are abundant and accessible and are evaluated in an expanded access program in elderly PD patients (PDPs) [17]. Umbilical cord/perinatal MSCs have high proliferative capacity and lower donor-age burden and are investigated broadly in regenerative medicine and neuroinflammation contexts [18].

Critical Translational Issues

The current challenges in the translation of cell therapy products are related to issues around donor variability, the effect of culture expansion, cryopreservation, potency assays, immunogenicity, and batch-to-batch consistency. These challenges are particularly pertinent since phase 2 PD data demonstrated mixed results across the dosing arms of the clinical studies in this area, and there have been recommendations regarding the need for functional potency verifications [19].

The proposed mechanisms of MSC therapy in PD are shown in Table 1.

**Table 1:** Proposed mechanisms of mesenchymal stem/stromal cell (MSC) therapy in Parkinson’s disease (PD) BBB: blood-brain barrier

Mechanistic domain	Biological action	Key molecular mediators	Relevance to PD pathophysiology	Supporting evidence
Immunomodulation	Suppression of pro-inflammatory cytokines; modulation of microglial activation	↓ TNF-α, ↓ IL-1β, ↓ IL-6; ↑ IL-10	Reduces chronic neuroinflammation contributing to dopaminergic neuron loss	[8,11]
Neurotrophic support	Secretion of growth factors promoting neuronal survival	BDNF, GDNF, VEGF	Supports nigrostriatal neuron survival and synaptic plasticity	[8,13]
Antioxidative effects	Reduction of oxidative stress and mitochondrial dysfunction	↑ antioxidant enzymes (SOD, catalase)	Mitigates oxidative injury implicated in PD progression	[8,13]
Antiapoptotic activity	Inhibition of neuronal apoptosis pathways	↓ caspase-3 activation	Protects dopaminergic neurons	[13,20]
α-Synuclein modulation	Reduction of aggregation and propagation	miRNA delivery via extracellular vesicles (EVs)	Targets the central pathogenic hallmark of PD	[2,10,14,15]
EV signaling	Transfer of miRNAs, proteins, and lipids across the BBB	miR-133b, miR-21, exosomal cargo	Enables cell-free therapeutic effects	[10,15,21,22]

Delivery Routes and Dosing Strategies

IV delivery: The use of IV infusions is readily achievable logistically and provides target systemic immune modulation, which could indirectly affect neuroinflammation by means of immune/brain crosstalk. Both a phase 1 (dose escalation) single IV infusion trial and a phase 2 (randomized) repeated IV infusion trial were conducted using allogeneic BM-MSCs administered via the IV route [6,7].

Intranasal and intracerebral routes: Intranasal administration is appealing because it allows medications to pass through the invading organism's blood-brain barrier and into the brain, while direct access to the brain via intracerebral/intra-arterial methods may increase the risk of complications from the procedure. Currently available clinical references for PD treatment delivery are still strongest in relation to IV (venous) methods; however, there are still limited, heterogeneous clinical resources related to non-IV (i.e., oral and intramuscular/intradermal) methods of delivery [11,13-15].

Mechanisms of action of MSCs in PD

Preclinical Evidence

MSCs have been shown to protect against toxin-induced (e.g., 6-hydroxydopamine (6-OHDA), 1-methyl-4-phenyl-1,2,3,6-tetrahydropyridine (MPTP), and rotenone) and certain genetic paradigms by reducing dopaminergic neuron degeneration, decreasing microglial activation/inflammatory cytokines, and improving behavioral status while also changing markers of oxidative stress [13,22,23]. A few studies indicate that MSCs can inhibit α-synuclein oligomerization and assist with innate repair mechanisms, but results are model-, cell source-, injection route-, injection timing-, and outcome measure-dependent [13,22,23]. A recent systematic review has indicated that MSCs show good promise; however, they demonstrate significant heterogeneity and create numerous translatability challenges [13,22,23].

Clinical Evidence in PD

Phase 1: single IV allogeneic BM-MSC infusion (dose escalation): A phase 1 study involving 20 patients with mild to moderate PD and another type of disease during a 12-month open-label study was published in 2020 [17].

Safety and tolerability: There were no serious infusion reactions; there were no immune responses against donor cells. The most common side effects included political and transient hypertension; one possibly serious side effect occurred in the patient who had an unrelated lymphocytic condition, who subsequently developed chronic lymphocytic leukemia (CLL).

Biological indicators: Higher doses demonstrated a reduction in several peripheral pro-inflammatory markers and an increase in BDNF at later endpoints.

Clinical indicators: The exploratory analysis of motor outcome data of the highest dosing group is supportive of safety and tolerability, but this study was not completed to determine efficacy.

Phase 2: repeated IV allogeneic BM-MSC infusions (randomized, placebo-controlled): An 18-week study compares three doses of living human stem cells compared to a placebo. There is an additional group that receives one dose of living human stem cells followed by two doses compared to the placebo group. The study will last for 88 weeks [18].

The primary outcome measured the number of subjects who achieved at least a 5-point improvement from baseline on the MDS-UPDRS (Movement Disorder Society Unified Parkinson's Disease Rating Scale) (Part III) after 62 weeks in the group receiving the three infusions of living human stem cells compared to the placebo group using a Bayesian analysis. The results show that there is greater than 70% probability of showing improvement in the three-infusion group [18].

There was a strong effect of the placebo condition in general, and therefore, the results of the second infusion group were not as profound compared to the placebo group. These results illustrate the variability and emphasize the need for potency and batch consistency assay development [18]. Adverse events associated with the infusions were reported as transient and minor in the abstract [18].

The summary table of clinical trials of MSC therapy in PD is shown in Table 2.

**Table 2:** Clinical trials of MSC therapy in Parkinson’s disease MSC: mesenchymal stem/stromal cell; BM-MSC: bone marrow-derived MSC; IV: intravenous; RCT: randomized controlled trial; MDS-UPDRS: Movement Disorder Society Unified Parkinson's Disease Rating Scale; AD-MSC: adipose-derived MSC

Study	Phase	MSC source	Dose & frequency	Route	Sample size	Primary outcome	Key findings
Brundin et al. [17]	Phase 1	Allogeneic BM-MSC	Single infusion (1-10 × 10⁶ cells/kg)	IV	n = 20	Safety & tolerability	Safe; no major immune reactions; exploratory motor improvement at higher doses
Vij et al. [18]	Phase 2 RCT	Allogeneic BM-MSC	10 × 10⁶ cells/kg × 3 infusions	IV	n≈45	MDS-UPDRS III responder rate	Met Bayesian primary endpoint; notable placebo response
Vij et al. [18]	Expanded access	Autologous AD-MSC	Multiple infusions over 18 weeks	IV	n = 10	Safety	Feasible; exploratory symptomatic improvement

Expanded access: autologous AD-MSCs in elderly PDPs: An intermediate-sized expanded access program looked at giving multiple IV infusions of autologous Hope Biosciences AD-MSCs to elderly PDPs (reported as n = 10). The age range is 76 through 95 [18]. The treatment duration is 18 weeks, immediately followed by the end-of-study evaluation of patients at 26 weeks [18]. Key findings (based on available report/abstract) include feasibility and safety focused on exploratory clinical efficacy endpoints [18]. Interpretation is limited because of the non-randomized design and small sample size.

Limitations and challenges

Evidence Synthesis

A systematic review of the clinical potential of MSCs in patients with PD found that MSC-based interventions offer potential but have limitations regarding the small number of participants tested, differences among the MSC types, their route of administration, and differences among the types of outcomes measured [13]. More recent mechanistic and clinical discussions identify additional opportunities and challenges for MSC therapies in PD by emphasizing their need for standardized and validated outcome measures and the development of biomarkers to assess their efficacy [10].

Quantitative meta-analyses were not conducted due to a significant lack of consistency among the studies. In fact, not only were there large variations across the design, characterization, sources, dosing strategies, and administration of MSC therapy, but the sources and standard definitions of outcomes were also very different. This lack of uniformity means that averaging data together would be methodologically unsound, as well as potentially generate misleading conclusions. Hence, a structured narrative synthesis approach has been created in order to evaluate which preclinical and clinical data exist related to MSC therapy and to orient such data with regard to the validity and directness of reference studies.

Safety considerations

Acute and Subacute Risks

Risks that could happen are infusion reactions, thromboembolic events (depending on product), infection transmissions (from allogeneic source/manufacturing), and immune sensitization. There was no evidence of immunogenicity signals (donor-specific antibodies) or serious adverse infusion-related events during phase 1 of the PD clinical trial [17]. Adverse events reported during phase 2 were mild and temporary as described in an abstract [24].

Theoretical and Long-Term Risks

Pro-fibrotic signaling and tumor-supportive microenvironment properties may also be of concern (dependent on context). Depending on age and other health issues that PDPs often face, long-term follow-up (especially those with declining health status) will be essential to track adverse events associated with MSC therapy [17].

Clinical Endpoints, Biomarkers, and Trial Design Challenges

Endpoint selection and placebo response: The core clinical outcome measure for most PD clinical trials is the MDS-UPDRS; minimal clinically important change (MCIC) thresholds provide a means to interpret whether the change reported is clinically relevant [25-27]. However, MSC trials can produce meaningful placebo responses and expectancy effects on outcome measurement, particularly when used in association with either invasive or "high-hope" interventions; this was previously identified in a commentary about phase 2 trial results [18].

Biomarkers to support mechanism and disease modification: To progress past symptoms, trials must include biomarkers demonstrating inflammation (cytokine panels); neurodegeneration (neurofilament light); imaging markers, such as DAT-SPECT/neuromelanin MRI; and, if feasible, α-synuclein measures. Phase 1 signals (i.e., TNF-α reduced and BDNF increased) are suggestive but need to be replicated in controlled studies [2,4].

Manufacturing Consistency and Potency Assays

The second phase of writing talks a lot about how much variation there is between different batches, and they give a recommendation to use functional potency tests (FPTs) to characterize product differences, which could be related to efficacy [9,20]. This recommendation fits in well with the general MSC literature regarding the importance of standardized reporting of information and robust criteria for product release.

Ethical and regulatory considerations

MSCs for PDPs are still experimental. The ethical considerations involve therapeutic misconception, “stem cell clinics” offering direct-to-consumer services, lack of typical regulation relative to other classes of medicine across the states, and patients who will be the most vulnerable due to progressive disabilities. Doctors should guide their patients through the process of trial participation because the scientific data surrounding the use of MSCs is still emerging, and enrolling in regulatory-approved clinical trials remains the safest route for access to MSC interventions [28].

Future directions

Future directions include multicenter trials with sufficient power, uniformity of stem cell products (particularly MSCs), and synchronous endpoints [28]. Assessment of potency-based dosing (linking in vitro quantitative functional assays of live tissue to in vivo response) should be associated with treatment arm performance metrics in the phase 2 study [18]. Use of multiple biomarkers to determine the mechanism of action (immunomodulation vs. neurotrophic support vs. EVs) should also be explored [28]. MSC-exosome or MSC-based therapies, as possible safer and more controllable “cell-free” biopharmaceutical products, should be investigated through extensive clinical evaluation [28]. Combined approaches (i.e., MSCs/exosomes + rehabilitation; combined MSCs/exosomes + neuromodulation therapy; combined MSCs/exosomes + targeted anti-inflammatory therapy) should also be evaluated using factorial designs [28].

## Conclusions

PD has seen progress transitioning from preclinical studies demonstrating promise toward early phase (i.e., traditional phase 1) clinical trials of human IV allogeneic BM-MSCs. Many IV allogeneic BM-MSC therapies have shown feasibility and short-term tolerability and have given initial signals of improvement in motor function in a controlled research environment. Despite these factors, the heterogeneity of the products used, the strong placebo effect, and the lack of full understanding of the mechanism of action have made it extremely difficult to draw definitive conclusions at this time. MSC-based therapies/interventions are considered investigational, and further progress will require established standards of manufacture, properly conducted potency testing, and rigidly enforced blinding and control protocols, as well as biologically informative biomarkers of therapy, to distinguish the symptomatic effects of therapy from disease-modifying effects.
